# Gastroduodenal Kaposi Sarcoma Without Cutaneous Lesions in an Immunosuppressed Patient

**DOI:** 10.7759/cureus.101282

**Published:** 2026-01-11

**Authors:** Ana Rita Ambrósio, Catarina Bico Filipe, Telma Costa Cabral, Hugo Pêgo, Ismenia Oliveira

**Affiliations:** 1 Internal Medicine, Hospital Beatriz Ângelo, Loures, PRT; 2 Nephrology, Hospital Beatriz Ângelo, Loures, PRT

**Keywords:** gastroduodenal involvement, gastrointestinal kaposi sarcoma, human herpesvirus 8, iatrogenic kaposi sarcoma, immunosuppressed patient

## Abstract

Kaposi sarcoma (KS) is an angioproliferative neoplasm that develops as a result of infection with human herpesvirus 8 (HHV-8). The disease most commonly appears as painless, nonpruritic, violaceous papules, plaques, or nodules on the skin, but it can also affect lymph nodes, mucosal surfaces, and internal organs.

A 53-year-old man from Guinea-Bissau with membranous nephropathy who was being treated with prednisolone and cyclophosphamide presented with abdominal pain, early satiety, anorexia, and fever. His symptoms emerged while he was taking antibiotics for a recent respiratory tract infection. Physical examination showed epigastric tenderness and bilateral ankle edema. Abdominopelvic CT revealed loss of the fat plane between the pancreatic head and the duodenum, along with multiple mesenteric and peripancreatic lymph nodes, some of which had necrotic changes. Serologic testing for HIV was negative, and both blood and mycobacterial cultures did not grow any organisms. Upper gastrointestinal endoscopy demonstrated lobulated, erythematous lesions in the gastric body, with similar changes in the duodenal bulb and the second portion of the duodenum. Biopsy showed a spindle-cell neoplasm, and immunohistochemistry confirmed KS, establishing the diagnosis of iatrogenic KS with gastroduodenal involvement. This HIV-negative case highlights that gastrointestinal KS should be considered in patients who are immunosuppressed due to medical therapy.

## Introduction

Kaposi sarcoma (KS) is a vascular neoplasm that is causally linked to human herpesvirus 8 (HHV-8) and occurs in four major clinical variants: classic, endemic, epidemic (HIV-associated), and iatrogenic (immunosuppression-related) [1-3]. These variants are classified mainly according to epidemiologic context and immune status, and clinical manifestations may overlap between variants. It typically presents with cutaneous lesions, but extracutaneous disease can affect lymph nodes, mucosal surfaces, and visceral organs [1-3]. Gastrointestinal involvement is often asymptomatic and can be easily missed [1,3,4]. Endoscopic findings vary, and diagnosis usually requires biopsy with confirmatory HHV-8 immunohistochemistry [1,3,4]. Gastrointestinal KS without skin lesions is rare, especially in HIV-negative patients, and may resemble infection or other malignancy on imaging and endoscopy [5,6].

Membranous nephropathy is one of the most frequent causes of nephrotic syndrome in adults and can be either primary or secondary to infections, medications, autoimmune disease, or malignancy [7,8]. Immunosuppressive therapy, although often required for disease control, increases the risk of opportunistic infections and immunosuppression-related neoplasms, including iatrogenic KS, highlighting the importance of thorough evaluation and management tailored to disease stage in immunocompromised patients [3,9,10]. We report the case of an HIV-negative, immunosuppressed patient with gastroduodenal KS in the absence of skin lesions who presented with abdominal symptoms and pancreaticoduodenal inflammatory changes on imaging.

This article was previously presented as a poster at the 31st National Congress of Internal Medicine (Portugal), held on May 22-25, 2025.

## Case presentation

The patient was A 53-year-old male from Guinea-Bissau who had been diagnosed with biopsy-proven membranous nephropathy (anti-PLA2R antibody-negative), with evaluation for secondary causes ongoing. He was receiving immunosuppressive therapy with oral cyclophosphamide (1.5 mg/kg/day) and prednisolone (0.25 mg/kg/day), along with trimethoprim-sulfamethoxazole for prophylaxis. A baseline abdominal CT scan performed during the etiologic workup of his nephropathy revealed splenomegaly and trace fluid along the right paracolic gutter, with mild mesenteric and perigastric fat stranding. His medical history included hypertension with poor adherence to therapy, dyslipidemia, hyperuricemia, and portal venous cavernomatous transformation. He reported remote episodes of malaria during travel, with the last episode occurring approximately ten years earlier, and had no known drug allergies.

Seven months after initiating immunosuppressive therapy, he developed fever and cough and was evaluated at a scheduled outpatient follow-up visit. At initial evaluation, laboratory tests showed worsening cytopenias, acute kidney injury, and elevated inflammatory markers (Table 1). Blood and urine cultures were obtained before antibiotic initiation, and were later found to be negative. Empiric therapy with amoxicillin/clavulanate and azithromycin was started, and cyclophosphamide was temporarily withheld. One week later, he was admitted for persistent fever and new gastrointestinal symptoms, including periumbilical abdominal discomfort, anorexia, early satiety, and a sensation of incomplete bladder emptying. He denied vomiting and reported preserved bowel movements but noted steatorrhea and intermittent hematochezia. He also reported an unintentional weight loss of approximately twenty kg over seven months, coinciding with the diagnosis of membranous nephropathy.

**Table 1:** Laboratory findings at initial evaluation and hospital admission Initial evaluation corresponds to the assessment at symptom onset (before antibiotic initiation), and hospital admission corresponds to the evaluation one week later. Reference ranges correspond to our institution’s laboratory; ‘Not available’ indicates that the test was not performed at that time point

Parameters (unit)	Initial evaluation	Hospital admission	Reference range
Hemoglobin (g/dL)	9.0	7.7	13-17
Leukocytes (10⁹/L)	3.9	3.52	4.5-11
Absolute neutrophil count (10⁹/L)	3.31	2.28	2.0-8.5
Absolute lymphocyte count (10⁹/L)	0.18	0.64	0.9-3.5
Platelets (10⁹/L)	235	323	150-450
Creatinine (mg/dL)	2.12	1.48	0.67-1.17
Urea (mg/dL)	108	58	16.6 – 48.5
C-reactive protein (mg/L)	115	102	<5
24-h urine protein (mg/24 h)	6231	Not available	0.00-140
Urinalysis-protein (mg/dL)	1000	1000	Not provided in the laboratory report
Urinalysis-erythrocytes (RBC) (/uL)	86	77	<17

**Figure 1:**
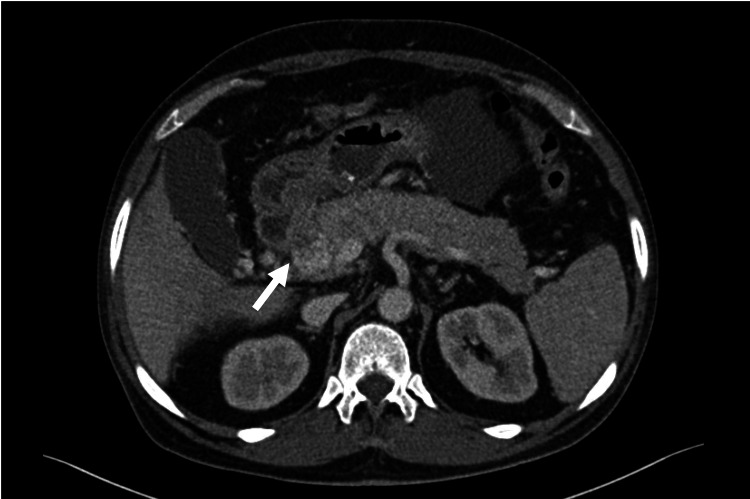
Abdominopelvic CT Abdominopelvic CT (axial). The image shows the obliteration of the fat plane between the pancreatic head and the duodenal sweep (arrow) with associated peripancreatic lymphadenopathy CT: computed tomography

Physical examination revealed no dermatologic lesions, but tenderness in the epigastric region was present. Abdominopelvic CT showed obliteration of the fat plane between the pancreatic head and the duodenal sweep (Figure 1) with adjacent inflammatory fat stranding, mild thickening of the ascending colon near the hepatic flexure, mesenteric root involvement, and peripancreatic lymphadenopathy (some with necrotic features), left inguinal lymphadenopathy with the largest node measuring 17 mm in short-axis diameter, and moderate perigastric and interloop fluid.

On hospital admission, laboratory evaluation revealed marked anemia, leukopenia with absolute lymphopenia, and elevated C-reactive protein, while renal function was improving compared with one week earlier (Table 1). Urinalysis showed significant proteinuria, mild hematuria, and hyaline casts (Table 1). Serum immunoglobulin G4 (IgG4) levels were within the normal range. Serologic testing for HIV was negative.

**Figure 2:**
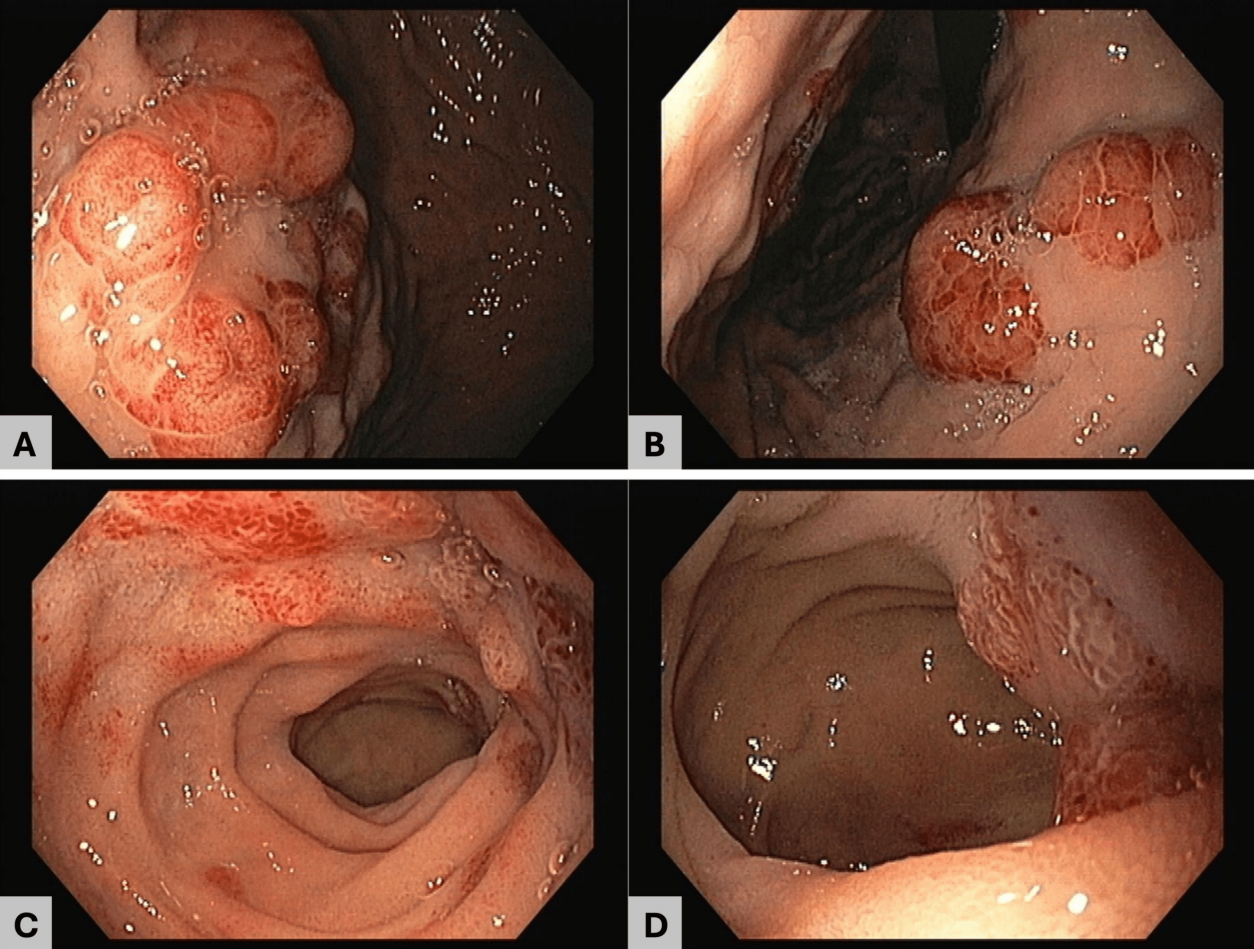
Upper gastrointestinal endoscopy findings (A, B) Gastric body showing raised, lobulated, hyperemic lesions with an infiltrative appearance. (C, D) Second portion of the duodenum (D2) demonstrating similar raised, lobulated, hyperemic lesions

**Figure 3:**
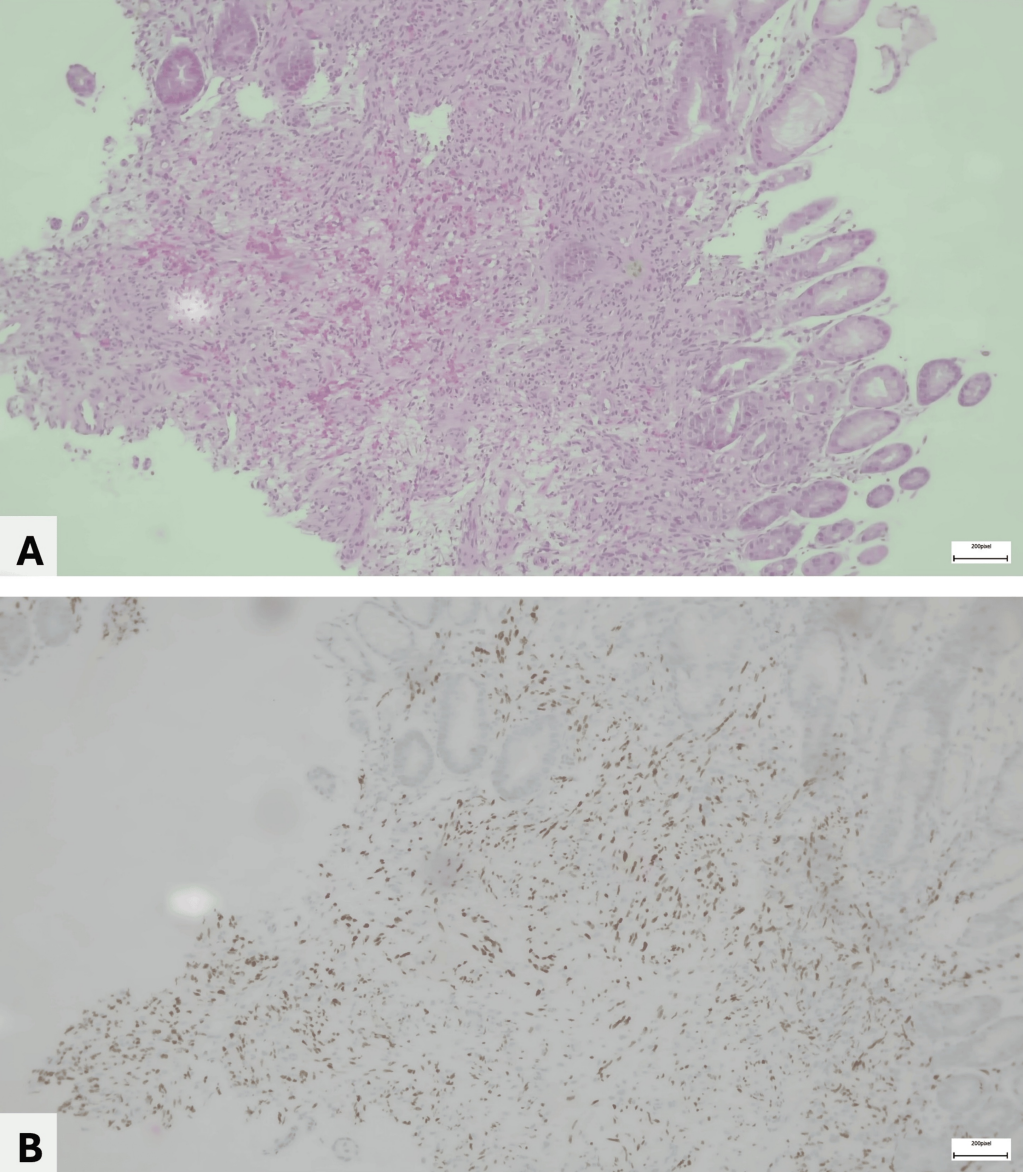
Duodenal biopsy histopathology (A) Hematoxylin–eosin stain, original magnification ×10, showing duodenal mucosal infiltration by a spindle-cell neoplasm with associated erythrocyte extravasation. (B) Human herpesvirus 8 immunohistochemical stain, original magnification ×10, demonstrating nuclear positivity in atypical spindle cells

Endoscopic ultrasound demonstrated septated perihepatic and perisplenic ascites and a necrotic-appearing lymph node near the hepatic margin. Upper endoscopy revealed raised, lobulated, hyperemic lesions with an infiltrative appearance in the gastric body, duodenal bulb, and second portion of the duodenum (Figure 2). Histopathologic examination showed a spindle-cell neoplasm with prominent angiogenesis (Figure 3A). Immunohistochemistry supported KS, with HHV-8 positivity (Figure 3B), confirming iatrogenic KS with gastroduodenal involvement. Mycobacterial studies, including nucleic acid amplification testing for *Mycobacterium tuberculosis*, were negative.

Abdominal MRI subsequently demonstrated superior mesenteric vein thrombosis with cavernomatous transformation and changes interpreted as probable acute-on-chronic pancreatitis. Therapeutic anticoagulation with low-molecular-weight heparin (tinzaparin) was initiated. After multidisciplinary discussion, management prioritized reduction of immunosuppression, with cyclophosphamide discontinued and prednisolone tapered to 5 mg/day under close clinical and radiologic surveillance. Additional laboratory evaluation identified prior exposure to the hepatitis B virus with low-level detectable HBV DNA, and antiviral therapy with entecavir was started.

**Figure 4:**
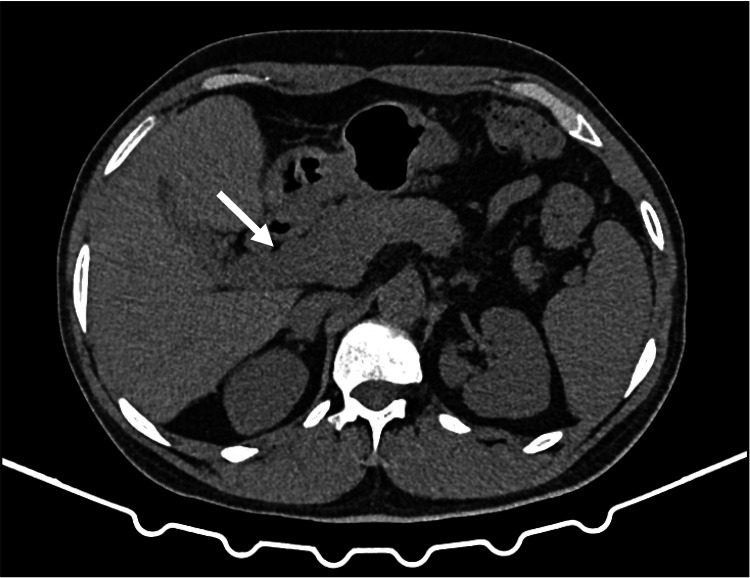
Follow-up abdominal CT (axial view) Axial CT image obtained on follow-up demonstrates improvement of the previously described pancreatic inflammatory changes (arrow) CT: computed tomography

**Figure 5:**
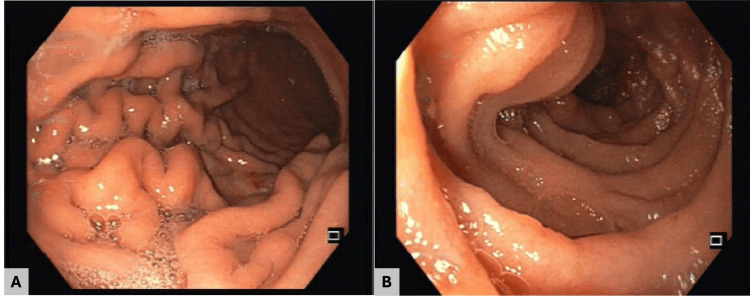
Surveillance upper endoscopy showing no residual or recurrent gastrointestinal Kaposi sarcoma (A) Gastric body: normal-appearing mucosa without lesions. (B) Second portion of the duodenum (D2): no mucosal lesions identified

On later reassessment, follow-up imaging showed improvement in the previously described pancreatic inflammatory changes (Figure 4), and the radiology report noted improving abdominal lymphadenopathy. Surveillance upper endoscopies demonstrated no residual or recurrent KS (Figure 5), and serial dermatologic examinations identified no suspicious cutaneous lesions throughout the disease course. At the 10-month follow-up, the patient remained under multidisciplinary care.

## Discussion

This report emphasizes practical diagnostic and management pitfalls of iatrogenic KS when it presents without cutaneous disease. Although gastrointestinal involvement in HIV-negative patients is uncommon and often asymptomatic, it may often be overlooked. When present, symptoms are usually nonspecific or reflect gastrointestinal bleeding with iron-deficiency anemia [2,4,5]. This presentation is clinically relevant because it can delay suspicion and prompt alternative diagnostic pathways [4-6].

A second diagnostic challenge in our patient was the radiologic and endoscopic mimicry of other conditions. Cross-sectional imaging showing loss of the fat plane between the pancreatic head and the duodenum, together with necrotic-appearing lymphadenopathy and ascites, raised concern for pancreatic malignancy, groove pancreatitis, or infection (including tuberculosis). Endoscopically, gastrointestinal KS may present with a wide spectrum of findings, including polypoid or nodular lesions, ulcerations, and infiltrative-appearing mucosal lesions, which can mimic more common benign or malignant pathology [4,5,11]. Overall, these findings reinforce the need for early histologic confirmation in equivocal presentations [2,4-6].

Histopathologic confirmation can be challenging because KS overlaps morphologically with a broad spectrum of spindle-cell lesions. In this context, immunohistochemistry can aid diagnosis, and HHV-8 latent nuclear antigen (LANA) is particularly helpful to support the diagnosis of KS and distinguish it from histologic mimickers, with complementary endothelial markers such as CD34 [1-3]. Therefore, biopsy with an appropriate immunohistochemical panel should be prioritized whenever KS is considered in the differential diagnosis [1-3].

Management in this case centered on balancing control of membranous nephropathy against removal of the immunosuppressive trigger for KS. While higher-risk membranous nephropathy may require sustained immunomodulatory therapy to prevent progressive kidney disease [7,8], corticosteroids and other immunosuppressive agents have been linked to iatrogenic KS, which may regress after dose reduction or withdrawal [3,9,10]. Current guidance emphasizes reducing or withdrawing immunosuppression when clinically feasible as a key therapeutic step, with systemic therapy generally reserved for aggressive, extensive, or disseminated disease [1,3]. In keeping with this approach, our patient's condition improved after the discontinuation of cyclophosphamide and steroid tapering, and subsequent endoscopies showed marked improvement consistent with remission.

Beyond the case-specific findings, this report underscores that the expanding use of immunosuppressive therapies may introduce unexpected diagnostic and therapeutic challenges, including iatrogenic KS in HIV-negative patients [9,10].

## Conclusions

This report illustrates that KS may develop as a complication of immunosuppression in HIV-negative patients and can present with nonspecific, multisystem manifestations that delay diagnosis. Clinicians should therefore maintain a high index of suspicion when encountering immunosuppressed patients with persistent constitutional or gastrointestinal symptoms and atypical imaging findings. Timely endoscopic biopsy with HHV-8 immunohistochemistry is central to confirming the diagnosis and guiding management. Careful adjustment of immunosuppression, supported by a multidisciplinary and individualized approach, can be an effective first-line strategy, while structured follow-up is important to monitor response and enable early detection of recurrence.

## References

[1] Lebbe C, Garbe C, Stratigos AJ (2019). Diagnosis and treatment of Kaposi's sarcoma: European consensus-based interdisciplinary guideline (EDF/EADO/EORTC). Eur J Cancer.

[2] Antman K, Chang Y (2000). Kaposi's sarcoma. N Engl J Med.

[3] Bettuzzi T, Lebbe C, Grolleau C (2025). Modern approach to manage patients with Kaposi sarcoma. J Med Virol.

[4] Carmo J, Marques SC, Bispo M, Pinto D, Chagas C (2017). Clinical and endoscopic features of gastrointestinal Kaposi sarcoma: a single-center Portuguese experience over the last decade. GE Port J Gastroenterol.

[5] Balachandra B, Tunitsky E, Dawood S, Hings I, Marcus VA (2006). Classic Kaposi's sarcoma presenting first with gastrointestinal tract involvement in a HIV-negative Inuit male--a case report and review of the literature. Pathol Res Pract.

[6] Akasbi Y, Awada A, Arifi S, Mellas N, El Mesbahi O (2012). Non-HIV Kaposi's sarcoma: a review and therapeutic perspectives. Bull Cancer.

[7] Ronco P, Beck L, Debiec H (2021). Membranous nephropathy. Nat Rev Dis Primers.

[8] Kidney Disease: Improving Global Outcomes (KDIGO) Glomerular Diseases Work Group (2021). KDIGO 2021 clinical practice guideline for the management of glomerular diseases. Kidney Int.

[9] Trattner A, Hodak E, David M, Sandbank M (1993). The appearance of Kaposi sarcoma during corticosteroid therapy. Cancer.

[10] Wu PJ, Sun CS, Kuo HT (2022). Iatrogenic Kaposi sarcoma of the small bowel in Crohn's disease following short-term use of immunomodulators: a case report and review of the literature. J Med Case Rep.

[11] Lee AJ, Brenner L, Mourad B, Monteiro C, Vega KJ, Munoz JC (2015). Gastrointestinal Kaposi's sarcoma: case report and review of the literature. World J Gastrointest Pharmacol Ther.

